# A district-level geospatial analysis of anaemia prevalence among rural men in India, 2019-21

**DOI:** 10.1186/s12939-023-02089-w

**Published:** 2024-01-19

**Authors:** Aditya Singh, Sumit Ram, Rakesh Chandra, Arabindo Tanti, Shivani Singh, Ananya Kundu

**Affiliations:** 1 Department of Geography, Banaras Hindu University, Varanasi, India; 2 School of Health System Studies, Tata Institute of Social Sciences, Mumbai, India; 3 Department of Public Health and Community Medicine, Central University of Kerala, Kasaragod, India; 4 Independent Researcher, Lucknow, India; 5 Department of Geography, University of Calcutta, Kolkata, India; 6 Girl Innovation, Research, and Learning (GIRL) Centre, Population Council, New York, USA

**Keywords:** Men’s anaemia, Men’s health, India, Spatial analysis, Spatial autocorrelation, Moran’s I

## Abstract

**Background:**

Despite its considerable impact on health and productivity, anemia among men has received limited attention. In a country as diverse as India, characterized by extensive geographic variations, there is a pressing need to investigate the nuanced spatial patterns of anemia prevalence among men. The identification of specific hotspots holds critical implications for policymaking, especially in rural areas, where a substantial portion of India’s population resides.

**Methods:**

The study conducted an analysis on a sample of 61,481 rural men from 707 districts of India, utilizing data from the National Family Health Survey-5 (2019-21). Various analytical techniques, including Moran’s I, univariate LISA (Local Indicators of Spatial Association), bivariate LISA, and spatial regression models such as SLM (Spatial Lag Model), and SEM (Spatial Error Model) were employed to examine the geographic patterns and spatial correlates of anaemia prevalence in the study population.

**Results:**

In rural India, three out of every ten men were found to be anemic. The univariate Moran’s I value for anaemia was 0.66, indicating a substantial degree of spatial autocorrelation in anaemia prevalence across the districts in India. Cluster and outlier analysis identified five prominent ‘hotspots’ of anaemia prevalence across 97 districts, primarily concentrated in the eastern region (encompassing West Bengal, Jharkhand, and Odisha), the Dandakaranya region, the Madhya Pradesh-Maharashtra border, lower Assam, and select districts in Jammu and Kashmir. The results of SLM revealed significant positive association between anaemia prevalence at the district-level and several key factors including a higher proportion of Scheduled Tribes, men in the 49–54 years age group, men with limited or no formal education, individuals of the Muslim faith, economically disadvantaged men, and those who reported alcohol consumption.

**Conclusions:**

Substantial spatial heterogeneity in anaemia prevalence among men in rural India suggests the need for region-specific targeted interventions to reduce the burden of anaemia among men in rural India and enhance the overall health of this population.

## Introduction

Anaemia is a haematological condition marked by a reduction in the haemoglobin concentration within the bloodstream, falling below established thresholds specific to age and gender [1]. This condition results in an inadequate number of red blood cells to effectively transport oxygen to the body’s tissues [2]. Iron deficiency, accounting for over 60% of cases globally, stands as the primary cause of anaemia [1]. However, deficiencies in other essential nutrients such as folate, vitamin B12, vitamin A, and exposure to lead can also lead to anaemia [3]. Genetic haemoglobin disorders such as thalassemia, blood loss resulting from trauma and gastrointestinal bleeding, malaria, tuberculosis, parasitic infestations also contribute to anaemia [4, 5].

Developing and underdeveloped nations confront numerous substantial health issues concerning nutrition, with anaemia being particularly persistent, intricate, and widespread. In 2019, approximately a quarter of the global population, nearly 1.8 billion individuals (world population 7.74 billion) experienced anaemia, with the majority of cases concentrated in developing countries [6]. Approximately 50 million Years Lived with Disabilities (YLDs) were reported across all anaemia causes, making anaemia the most prevalent impairment for both the sexes combined globally [7]. The age-standardized point prevalence of anaemia was notably high in South Asia, reaching 41.6%, a figure quite comparable to rates observed in western Sub-Saharan Africa (SSA). Within South Asia, India grapples with a substantial public health challenge related to anaemia, contributing to around one-third of all global cases of anaemia (583 million cases in 2019) [8]. This high prevalence is striking given India’s access to a diverse range of foods, including grains, fruits, vegetables, and other nutritional resources [9].

Anaemia is discovered to have a number of underlying negative impacts. Affecting over half the population in almost all age groups of women and children, anaemia can have devastating consequences on human health and result into poor socioeconomic development [10]. One significant concern is iron deficiency anaemia, which obstructs cognitive development and growth in children [11]. Maternal anaemia also carries serious implications for pregnant women and their newborns, such as low birth weight [12], preterm birth, stillbirth, postpartum haemorrhage [13] and has the potential to cause even maternal mortality [13, 14]. While studies on anaemia in low and middle-income countries (LMICs) have predominantly cantered on women of reproductive age (WRA) and children, there exists a noticeable gap in the research focusing on anaemia in men. Nevertheless, it is established that anaemia is prevalent among men and can yield substantial consequences, causing fatigue, concentration difficulties, and lethargy. These consequences not only erode the quality of life for affected individuals but also have detrimental implications for the economic productivity of a nation [15, 16]. Evidence suggests that iron deficiency anaemia stands is a significant contributor to the overall burden of disease among men across all states and union territories (UTs) of India [17].

Several prior studies conducted in African and South Asian countries have emphasized the importance of investigating spatial heterogeneity in anaemia. For instance, a recent study conducted in Nepal delved into the factors linked to anaemia among WRA and employed multilevel modelling and spatial analysis tools to identify geographical clustering in anaemia prevalence [18]. Additionally, a recent study focusing on SSA by Correa-Agudelo et al. (2021) assessed the contribution of risk factors linked to anaemia in WRA and mapped the high burden of anaemia (19) while Roberts et al. (2020) delineated spatial inequalities in anaemia among children between the ages of 6 and 59 months in four SSA countries Kenya, Malawi, Tanzania, and Uganda (20). In India, Sharma et al. (2020) highlighted the spatial heterogeneity in anaemia among children using data from the fourth round of the National Family Health Survey (NFHS-4) (21). However, all of the studies were limited to WRA and children.

**Fig. 1:**
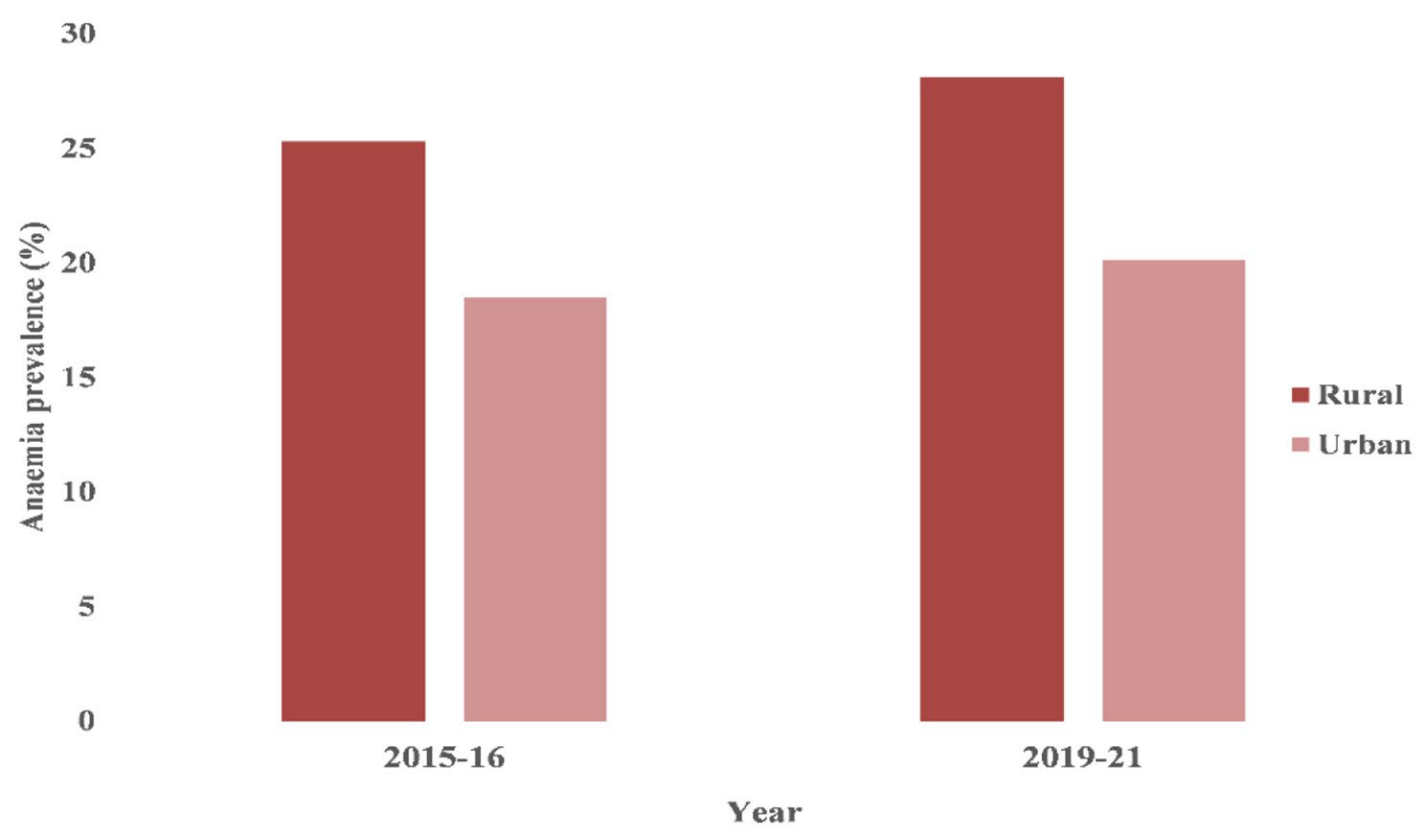
Prevalence of anaemia among men India by place of residence, NFHS-4 (2015-16) and NFHS-5 (2019-21)

Recent estimates from a nationally representative survey in India revealed that approximately a quarter of men aged 15–49 years in the country were estimated to be anaemic, with an even higher prevalence among rural men at 28.1% [19]. There has been a noticeable, albeit modest, increase in the prevalence of anaemia among the rural male population over between 2015-16 and 2019-21 **(**Fig. 1**)**. Prevalence of anaemia in men at aggregated levels is only of limited importance from action perspective in a large and heterogeneous country like India. Variations in dietary habits, lifestyles, and socioeconomic statuses in different regions of India might have resulted in differing rates of anaemia among men, especially in rural areas [20, 21]. A detailed state- and district-level spatial analysis of anaemia prevalence is therefore crucial to inform relevant health policy and health service interventions aimed to address the issue.

The existing evidence regarding the geographical aspects of men’s anaemia in rural India is notably scarce. A previous study conducted by the authors focused primarily on investigating the factors associated with anaemia among rural Indian men, with only a cursory mention of its geographic distribution [22]. A comprehensive spatial analysis goes beyond the mere mapping of anaemia prevalence. It entails the detection and measurement of spatial dependence, often referred to as spatial autocorrelation [23]. This analysis also incorporates cluster and outlier identification to pinpoint areas with high and low values of the variable of interest. Additionally, spatial regression models were employed to address spatial dependence in the data, as simple regression models become invalid in such contexts. To date, none of the available studies has ventured into conducting such a thorough state and district-level analysis of the geographical patterns of anaemia among men in rural India.

This study, therefore, aims to investigate the state and district-level spatial heterogeneity of anaemia among men in rural India. It identifies the specific hotspots where anaemia prevalence is concentrated. Additionally, the study employs spatial regression models to uncover the factors associated with anaemia prevalence at district level. This analysis yields essential insights for crafting location-specific interventions and well-informed strategies to alleviate the overall burden of anaemia among rural Indian men. Ultimately, this research endeavours to contribute significantly to the reduction of the national anaemia burden among men and enhance health outcomes in rural communities.

## Data and methods

### Data source

The study relied on the data from the most recent round of the National Family Health Survey, conducted between 2019 and 2021, commonly referred to as NFHS-5. This survey covered a number of issues including health and nutrition of men, women, and children. It collects data from all states and union territories (UTs) within India, as well as 707 individual districts. A total of 101,839 men and 724,115 women ranging in age from 15 to 54 years and 15 to 49 years respectively, from 636,699 households were surveyed. The response rate for men and women was 92% and 97%, respectively [19]. More about the survey methodology is available in the NFHS-5 national report.

The NFHS-5’s clinical, anthropometric, and biochemical (CAB) component was used to collect data on malnutrition, anaemia, hypertension, elevated blood glucose, waist and hip size, Vitamin D3, HbA1c, and the presence of malaria parasites. The Biomarker Schedule included measures of the height, weight, waist, and hip circumferences as well as haemoglobin levels of individuals [19].

### Sample selection

Out of the 111,179 eligible men aged 15 to 54 years, 101,839 who were usual residents and had spent the night before the survey in their residences were selected for the state module. Among these selected men, 92,820 consented to have their haemoglobin levels checked. However, 31,338 of these consenting individuals were exclused from the study sample because they resided in urban areas. As a result, the present research was restricted to 61,482 men who lived in rural areas.

### Anaemia testing

In NFHS-5, iInvestigators collected blood specimens for anaemia testing from eligible men aged 15–54 years. Prior to the collection, respondents’ consent for the test was obtained. The blood samples were obtained through a finger prick method and were collected in micro cuvettes. On-site analysis of haemoglobin levels was performed using a battery-operated portable HemoCue Hb 201 + analyser. Additionally, haemoglobin levels were adjusted for altitude in enumeration areas situated at altitudes above 1,000 m to account for potential altitude-related variations [19].

### Outcome variable

The outcome variable was prevalence of anaemia i.e., whether a man was anaemic or not at the time of the survey. Men were classified as ‘not anaemic’ if their haemoglobin concentration was 13.0 g/dL or higher, mildly anaemic if it ranged from 12.0 to 12.9 g/dL, moderately anaemic if it fell between 9.0 and 11.9 g/dL, and severely anaemic if it was below 9.0 g/dL, in accordance with World Health Organization (WHO) guidelines [24]. In this analysis, a binary variable was established, classifying men with haemoglobin levels below 13 g/dL as ‘anaemic’ [1], and those with levels above 13 g/dL as ‘not anaemic’ (0).

### Exposure variables

A comprehensive set of factors associated with the prevalence of anaemia among men were chosen for inclusion in this study [18, 22, 25, 26]. The selection of these factors was guided by prior research findings and data availability. These factors encompass a wide range of variables, including age, educational attainment, caste, religion, household wealth, Body Mass Index (BMI), alcohol consumption, and smoking habits. Table 1 provides the description of the independent variables selected for this study and Table 2 provides the descriptive statistics of these variables.

**Table 1:** Description of the variables

Variables	Description	Source
Anaemia prevalence (%)	Percentage of rural men found anaemic in a district	NFHS-5, 2019-21
Older men (%)	Percentage of men aged 49–54 years in a district	NFHS-5, 2019-21
No education (%)	Percentage of men with no formal education in a district	NFHS-5, 2019-21
Scheduled Tribe (%)	Percentage of men from the Scheduled Tribe category in a district	NFHS-5, 2019-21
Muslim (%)	Percentage of Muslim men in a district	NFHS-5, 2019-21
Poorest households (%)	Percentage of men belonging to the poorest quintile of the households in a district	NFHS-5, 2019-21
Underweight (%)	Percentage of men found underweight (BMI < 18.5 kg/m^2^) in a district	NFHS-5, 2019-21
Alcohol (%)	Percentage of men who consumed alcohol daily or weekly in a district	NFHS-5, 2019-21
Smoking (%)	Percentage of men who smoked daily or weekly in a district	NFHS-5, 2019-21

**Table 2:** Descriptive statistics of variables, India, 2019–2021

Variables	Mean (%)	SD (%)	Minimum (%)	Maximum (%)
Anaemia prevalence (%)	25.10	12.75	0	80.47
Older men (%)	8.30	3.79	0	23.24
No education (%)	12.80	8.94	0	65.44
ST (%)	21.32	30.25	0	100
Muslim (%)	9.55	18.11	0	100
Poorest households (%)	22.56	20.35	0	83.92
Underweight (%)	15.79	8.26	0	40.56
Alcohol (%)	70.33	20.29	0	100
Smoking (%)	43.57	18.19	0	83.08

Note: SD: Standard Deviation, ST: Scheduled Tribe

### Statistical analysis

Geographical phenomena typically exhibit non-random variations, displaying a tendency to cluster and concentrate in specific geographic areas. Governed by the first law of geography, objects in proximity tend to share more similarities and interactions than those located at a greater distance. This inclination toward spatial clustering in data is referred to as spatial dependency or spatial autocorrelation (SA) [23]. SA has a significant effect on the accuracy of classical statistics. In conventional statistics, the observations are assumed to be independent of each other. However, in the presence of spatial autocorrelation (SA), this conventional statistical assumption is not valid. This is precisely why it is crucial to perform tests for both global and local SA before conducting any complex statistical analysis involving spatial data.

Those measures that estimate SA by a single value for the entire study area are called ‘global’ measures. Among the various diagnostic measures for global SA, Global Moran’s I index is a popular measure. It assesses the presence of spatial patterns (clustering) within the data and determines the statistical significance of these patterns. This assessment is typically carried out using the following formula:

$$ Global\ Moran's\ I=\frac{n}{\sum _{i}^{n}\sum _{j}^{n}{w}_{ij}}\frac{\sum _{i}^{n}\sum _{j}^{n}{w}_{ij}\left({x}_{i}- \stackrel{-}{x}\right)\left({x}_{j}- \stackrel{-}{x}\right)}{\sum _{i}^{n}{\left({x}_{i}- \stackrel{-}{x}\right)}^{2}}$$

Where, n is the number of the spatial features; $$ {x}_{i}$$ is the attribute value of feature *i*, (remember that a variable is also called attribute in the spatial analysis context); $$ {x}_{j} $$is the attribute value of feature *j*; $$ \stackrel{-}{x}$$ is the mean of this attribute; $$ {w}_{ij}$$ is the spatial weight between feature *i* and *j*; $$ \sum _{i}^{n}\sum _{j}^{n}{w}_{ij}$$ is the aggregation of all spatial weights.

The value of Moran’s I varies between − 1 (perfect dispersion) and 1 (perfect clustering). A value of zero denotes a random geographical pattern. A positive (negative) Moran’s I value denotes positive (negative) spatial autocorrelation which means that points with similar attribute values (in our case district anaemia prevalence in %) are clustered together on the map (clustering), whereas negative spatial autocorrelation implies that points with dissimilar attribute values are clustered together (dispersion) [23, 27]. A Moran’s I value close to zero suggests that there is no significant spatial autocorrelation, implying a random or uniform distribution of values across the study area.

‘Global’ measures such as Moran’s I though indicate presence of clustering in data, they do not provide information about the location of clusters on the map. In order to determine the location of clusters on the map, one should calculate Local Indicators of Spatial Association (LISA) [23], such as “Local” Moran’s I, which is also known as cluster and outlier analysis. Cluster and outlier analysis output produces a map showing two types of statistically significant clusters: high-high clusters and low-low clusters. The high-high clusters represent areas (in our case districts) where a particular variable of interest (in our case anaemia) has both high values and is surrounded by high value neighbouring areas (districts). A low-low cluster is specific geographic area where a particular variable or attribute has low values and is surrounded by similarly low value neighbouring areas. It also detects two types of outliers: high-low (high-prevalence district surrounded by low-prevalence districts) and low-high (low-prevalence district surrounded by high-prevalence districts).

While univariate Moran’s I is designed to assess the spatial autocorrelation of a single variable by examining the correlation between a variable on the x-axis and its spatial lag (which pertains to the same variable) on the y-axis, bivariate Moran’s I serves the distinct purpose of evaluating the spatial dependence between two separate variables, where one variable is represented on the x-axis, and the spatial lag of a different variable is shown on the y-axis. Bivariate Moran’s I is used in investigating and quantifying spatial relationships and dependencies between two distinct variables measured at the same locations. Its utility lies in determining whether there is a spatial association or clustering between an outcome variable (such as anaemia prevalence) and the corresponding values of an exposure variable (such as age, BMI, etc.) across a defined geographic area. Additionally, the analysis includes the calculation of bivariate LISA, which is then used to create cluster and outlier maps to aid in identifying instances of spatial co-clustering between anaemia and selected exposure variables [23].

Moreover, a series of regression models were applied to investigate the factors associated with anaemia prevalence among men at the district level in India. Challenges arise when sample data observations have a locational or spatial component, manifesting in two issues: (a) spatial dependence, and (b) spatial heterogeneity. Conventional regression models, like the Ordinary Least Squares (OLS) linear regression model, overlook these concerns and are thus deemed unsuitable for spatial data analysis. The OLS regression assumes independent observations and normally distributed residual errors. However, this assumption is compromised in the presence of spatial autocorrelation (SA), leading to underestimated standard errors, overstated statistical significance, and a potential misinterpretation of relationships. Therefore, when SA is present, the use of an OLS model becomes invalid. Instead, it is imperative to consider and test spatial regression models such as the Spatial Lag Model (SLM) and Spatial Error Model (SEM). These models account for spatial dependencies, ensuring the robustness of the analysis and mitigating the limitations of traditional regression approaches.

An OLS model can be expressed as:

$$ y=\alpha +\beta X+ \epsilon $$

Where $$ y$$ denotes the outcome variable (anaemia prevalence), X symbolises the vector of predictor variables, α the model intercept, and β is the corresponding coefficient vector [28].

The Spatial Lag Model (SLM) serves as an extension of Ordinary Least Squares (OLS), incorporating a spatially lagged variable (spatial lag term) alongside the existing independent variables on the right-hand side of the equation. This spatially lagged variable is computed based on the dependent variable and represents the weighted sum of neighbouring values of the dependent variable (*y*) for each location within its neighbourhood. This model controls for SA in the dependent variable. This model can be expressed as:

$$ y=pWy+Xb+u$$

Where *y* is the dependent variable (anaemia prevalence in our case), *Wy* is the spatial lag term (spatially lagged anaemia prevalence), *p* is the coefficient associated with spatially lagged variable, *X* is the independent variable(s), *b* is the coefficient associated with *X*, and *u* is representing the error term in the model.

On the other hand, SEM is used when there is a belief that the residuals (errors) from an OLS regression exhibit spatial autocorrelation, meaning that they are correlated with the errors in neighbouring locations [28]. A typical SEM regression equation is written as:

$$ y=Xb+u$$

Where, *y* is the dependent variable, *X* is the independent variable(s), *b* is the coefficient associated with *b*. u is the error term with SA. It is defined as $u=\lambda Wu+\epsilon$, where W is the spatial weights matrix and $\lambda$ is the spatial autoregressive parameter, and ε represents the uncorrelated errors.

To decide which model (SLM or SEM) was more appropriate, Akaike Information Criterion (AIC) value was used. ArcMap version 10.5 [29], GeoDa [30] and Stata-16 [31] software applications were used for analysing the data and creating the maps for this research article.

## Results

### Geographical variations in anaemia among men

**Fig. 2:**
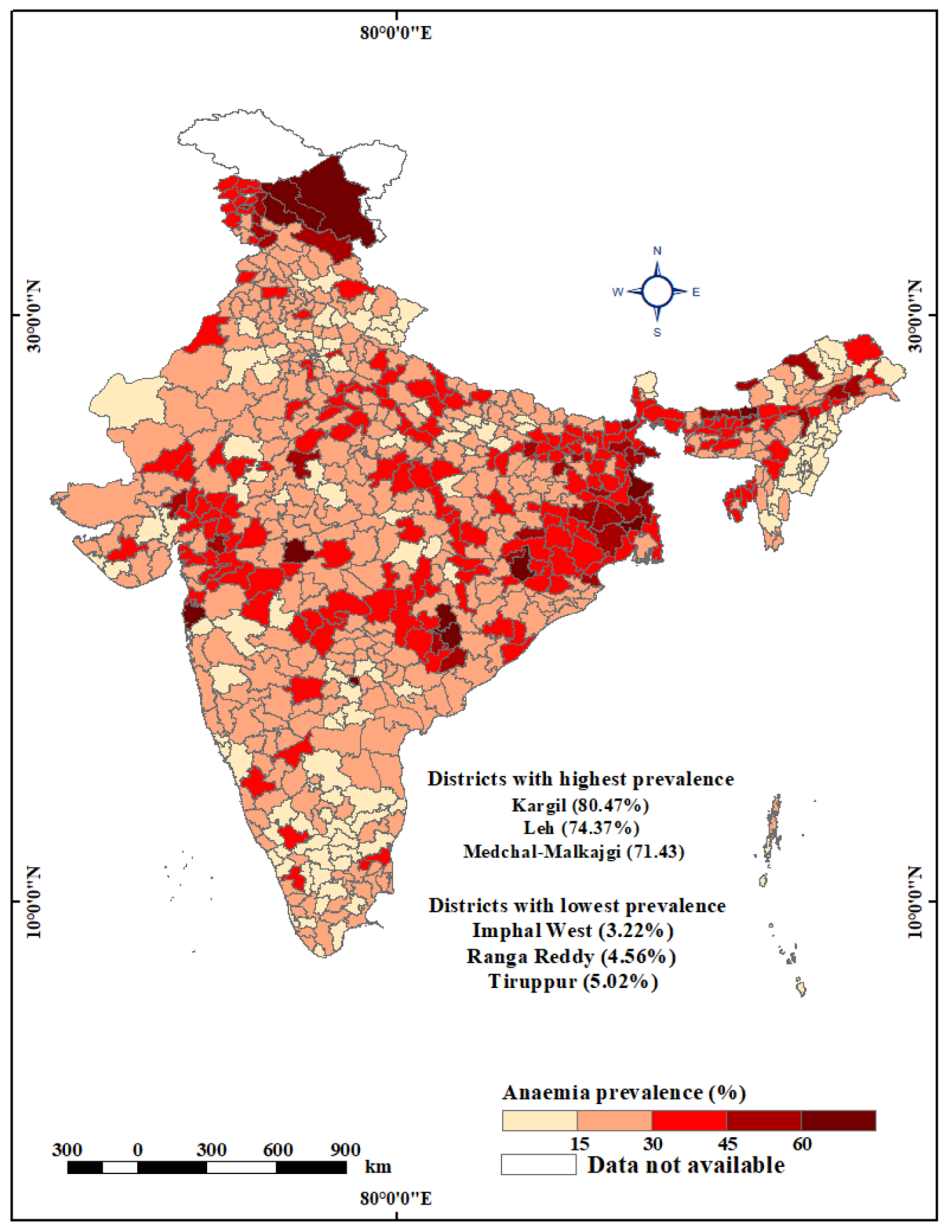
Map showing spatial variation in prevalence of anaemia among rural men across 707 districts in India, NFHS-5, 2019-21 (Map created by authors. The base layer is a free GIS file taken from https://spatialdata.dhsprogram.com/boundaries/#view=table&countryId=IA for national and sub-national boundaries)

Figure 2 illustrates stark spatial disparities in anaemia prevalence among rural men across India’s 707 districts, with a notable contrast between the eastern and southern regions of the country. This study revealed that close to 30% of men in rural India suffered from anaemia in 2019-21. Among the 707 districts studied, 12 districts emerged with anaemia prevalence rates exceeding a concerning threshold of 60%. These areas signal a critical health issue demanding immediate attention. An additional 36 districts exhibited anaemia prevalence rates ranging from 45 to 60%, highlighting a substantial health challenge, though somewhat less severe. These high-prevalence districts were predominantly clustered in specific states, namely West Bengal, Odisha, Chhattisgarh, and Assam, and union territories including Jammu and Kashmir and Ladakh. Among the 707 districts analysed, 177 districts fell within the range of 30-45% anaemia prevalence, while an even larger number, 336 districts, had prevalence rates between 15% and 30%. In contrast, the lowest anaemia prevalence, less than 15%, was observed in 146 districts, primarily located in the southern states of Tamil Nadu and Karnataka, as well as in the north-eastern states of Nagaland and Mizoram, and the northern states of Uttarakhand and Himachal Pradesh.

### Results of spatial autocorrelation and hotspot analysis

**Fig. 3:**
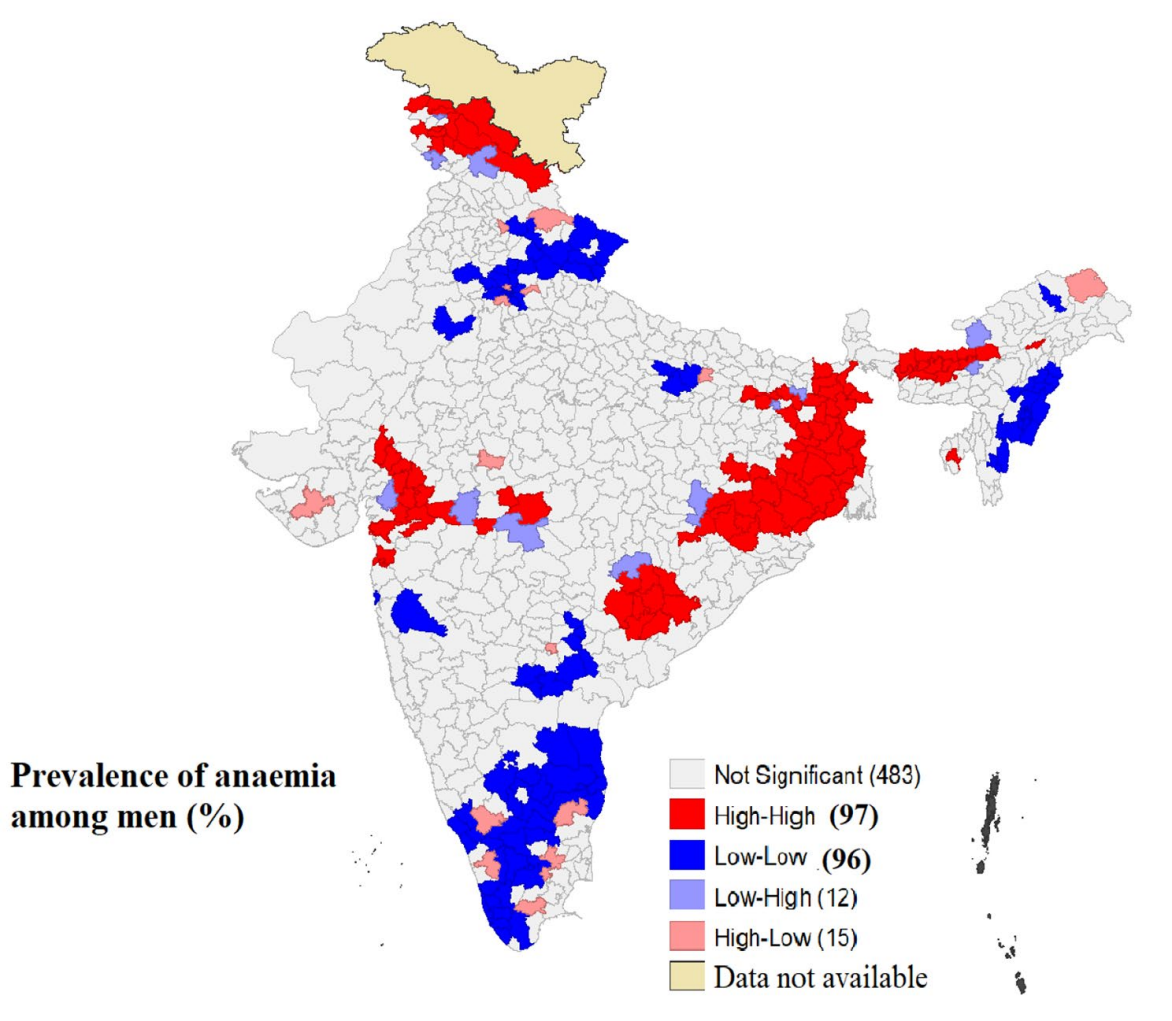
Univariate LISA map showing spatial clustering of anaemia among rural men across 707 districts in India, NFHS-5, 2019-21 (Map created by authors. Base layer is a free GIS file from https://spatialdata.dhsprogram.com/boundaries/#view=table&countryId=IA for national and sub-national boundaries)

Table 3 shows the Moran’s I values for the outcome (anaemia) and the exposure variables. The univariate Moran’s I value for anaemia was 0.66, indicating a substantial degree of spatial autocorrelation across districts in India. Univariate LISA cluster map for anaemia (Fig. 3) showed that high-high clusters or ‘hotspots’ of anaemia (districts with above-average anaemia prevalence sharing boundaries with neighbouring districts that had also above-average values) were found in 97 districts. The majority of these hotspots were located in five clusters, including the eastern part of India (West Bengal, Jharkhand, and Odisha), the Dandakaranya area, the Madhya Pradesh-Maharashtra border, lower Assam, and a few districts of UT Jammu and Kashmir. Low-low clusters or ‘cold spots’ of anaemia were reported (districts with below average prevalence of anaemia surrounded by districts with below average prevalence) in 96 districts. Southern India (districts in Tamil Nadu, Andhra Pradesh, and Kerala), Uttarakhand, and the mountainous regions of Nagaland and Manipur were the main cold spots for anaemia.

**Table 3:** Moran’s I statistic showing spatial dependence of anaemia among rural men and different independent variables across districts of India, NFHS-5, 2019-21 (*N* = 707)

Variables (District level)	Univariate		Bivariate (Anaemia prevalence and correlates)	
	Moran’s I	*p*-value	Moran’s I	*p*-value
Anaemia prevalence (%)	0.53	0.001	NA	NA
Older men (%)	0.21	0.001	0.14	0.001
No education (%)	0.54	0.001	0.31	0.001
ST (%)	0.70	0.001	0.23	0.121
Muslim (%)	0.60	0.001	0.28	0.001
Poorest wealth status (%)	0.74	0.001	0.35	0.001
Underweight (%)	0.51	0.001	0.16	0.172
Alcohol (%)	0.55	0.001	0.17	0.001
Smoking (%)	0.67	0.001	0.26	0.001

Note: NA: Not applicable; SC: Scheduled Tribe

Among the determinants, the value of Moran’s I statistic was highest for proportion of poorest men (0.74). It was also very high for ST men (0.70) followed by men who smoke (0.67), Muslim men (0.60), men consuming alcohol daily (0.55), men with no education (0.53) and underweight men (0.50). Significant geographic autocorrelation is evident from the high Moran’s I statistics for the variables used in the analysis. On the other hand, percentage of older men showed low spatial autocorrelation (0.21).

### Spatial association of anaemia prevalence with men’s background characteristics

**Fig. 4:**
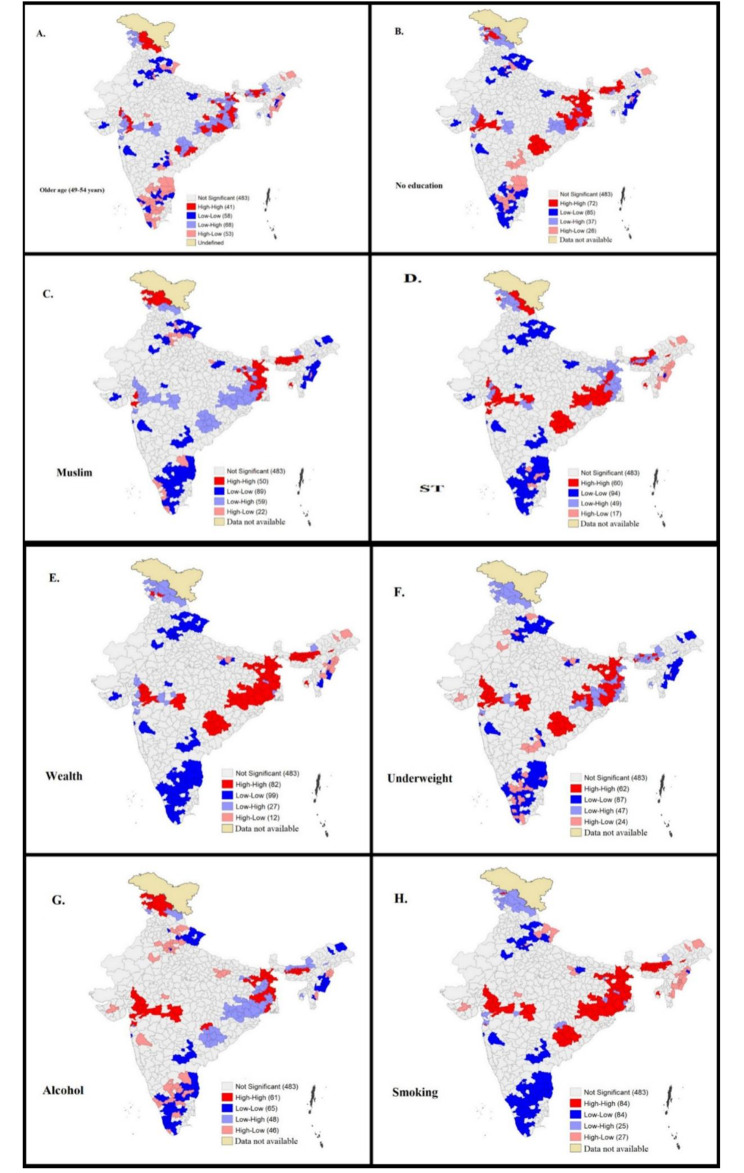
Bivariate LISA maps showing spatial association between anaemia among rural men and independent variables across 707 districts in India, NFHS-5, 2019-21 (Map created by authors. The Base layer is a free GIS file downloaded from https://spatialdata.dhsprogram.com/boundaries/#view=table&countryId=IA for national and sub-national boundaries)

The Bivariate Moran’s I value for anaemia and its major correlates is given in Table 3. Figure 4A depicts that 41 districts constitute the hotspots (high levels of anaemia and high percentage of older men) while 58 districts form the cold spots (low levels of anaemia and low percentage of older men). The hotspot districts were located mainly in West Bengal, Odisha, Assam, Jammu and Kashmir. In comparison, the cold spot districts sprawled mainly over Jammu and Kashmir, Uttarakhand, NCR (National Capital Region) and haphazardly in south Indian districts.

Figure 4B shows that high anaemia prevalence was statistically correlated with high percentage of uneducated men in 72 districts (hotspots), located mainly in eastern India, northern Maharashtra, the Dandakaranya region and Assam. On the other hand, low anaemia was significantly correlated with low percentage of uneducated men in 85 districts (cold spots) in southern India, Mizoram, Nagaland, and Uttarakhand.

Figure 4C presented high-high clusters between percentage of Muslim men and anaemia prevalence in 50 districts of West Bengal, Assam, and UT Jammu and Kashmir. On the other hand, low-low clusters between the same were found in 89 districts in Kerala, Tamil Nadu, Karnataka, some parts of Andhra Pradesh, Nagaland, Mizoram, and Uttarakhand. High percentage of ST population and high anaemia prevalence were correlated in 60 districts that belonged to Odisha, Chhattisgarh, and northern part of Maharashtra while 49 districts showed correlation between low percentage of ST men and low prevalence of anaemia. These districts were located mainly in southern India (Kerala, Tamil Nadu, Karnataka), Uttarakhand and Haryana **(**Fig. 4D**)**.

The map of spatial correlation between percentage of poorest men and anaemia (Fig. 4E) also revealed some interesting spatial patterns. High prevalence of anaemia was significantly correlated with high percentage of men belonging to the poorest households in 82 districts. These districts belonged to West Bengal, Bihar, Jharkhand, Odisha, Chhattisgarh, Assam, and northern Maharashtra while the 89 cold spot districts (low percentage of poorest men and low anaemia prevalence) encompassed mainly in southern Indian states, Uttarakhand, and Jammu and Kashmir.

As far as the correlation between BMI and anaemia is concerned, Fig. 4F exhibits those 72 districts in Bihar, West Bengal, northern Maharashtra, western Madhya Pradesh that formed high-high clustering compared to 85 districts constituting low-low clustering in south Indian states, Jammu and Kashmir, Uttarakhand, Nagaland, and Mizoram.

61 out of 707 districts forming hotspots for percentage of men who consumed alcohol and anaemia prevalence were located in West Bengal, Bihar, Assam, north Maharashtra, and UT Jammu and Kashmir (Fig. 4G). Figure 4H depicted that percentage of men who smoked and high prevalence of anaemia showed statistically significant correlation in 84 districts in West Bengal. Bihar, Jharkhand, Odisha, Chhattisgarh.

### Results of regression models

Table 4 presents the estimates from OLS, SEM and SLM exhibiting spatial association between anaemia prevalence and its correlates. Based on the AIC values, the SLM was found to the best fit model as it had the lowest AIC value among the three models. The AIC value for SLM was 5258.39 (*p* < 0.001), while the lag coefficient (Ρ) was 0.488 (*p* < 0.001) and the adjusted R^2^ value 0.442.

**Table 4:** Results of ordinary least squares, spatial lag, and spatial error regression models showing factors associated with anaemia among rural men, India, NFHS-5, 2019-21

	OLS Model	SLM	SEM
Independent variables	Coefficient (*p*-value)	Coefficient (*p*-value)	Coefficient (*p*-value)
Older men (%)	0.204 (0.064)	0.256 (0.008)	0.250 (0.017)
No education (%)	0.235 (0.000)	0.192 (0.000)	0.254 (0.000)
ST men (%)	0.024 (0.147)	0.016 (0.286)	0.010 (0.631)
Muslim men (%)	0.151 (0.000)	0.096 (0.000)	0.075 (0.014)
Poorest households (%)	0.150 (0.000)	0.072 (0.008)	0.109 (0.002)
Underweight (%)	0.042 (0.519)	0.001 (0.988)	0.003 (0.959)
Alcohol (%)	0.081 (0.000)	0.069 (0.001)	0.100 (0.000)
Smoking (%)	0.063 (0.038)	0.046 (0.089)	0.083 (0.011)
N	708	708	708
Lag coefficient (Ρ)		0.488 (0.000)	
Lag coefficient (λ)			0.530 (0.000)
AIC	5387.57	5258.39	5263.81
R- squared	0.293	0.442	0.442

Notes: OLS: ordinary least squares, SLM: spatial lag model, SEM: spatial error model

In the SLM, the analysis uncovers significant associations between several factors and anaemia prevalence in India’s districts. Specifically, the proportion of older men, men with no education, Muslims, poorest men, and men consuming alcohol were identified as spatially and statistically correlated with anaemia prevalence at district level. A 10-point increase in the percentage of older men was linked to a 2.6-point rise in anaemia prevalence, suggesting a higher prevalence among older individuals. Similarly, a 10-point increase in the percentage of men with no education was associated with a 1.9-point increase in anaemia prevalence, highlighting the impact of education level. Religion also played a role, with a 10% increment in the Muslim male population contributing to a 1% increase in anaemia prevalence. Wealth status was found to be a significant factor, as a 10% rise in the population of poorest men results in a 0.7% increase in anaemia prevalence. Finally, men’s alcohol consumption was associated with anaemia, as a 10% increase in alcohol consumption corresponds to a 0.7% increase in anaemia prevalence.

## Discussion

The exploration of men’s anaemia remains relatively limited, despite its potential significant impact on the health, well-being, and productivity of men. It is therefore crucial to develop a comprehensive understanding of this issue to devise measures that aid in reduction of anaemia among men. In this study, we undertook a comprehensive analysis of spatial variations in anaemia prevalence at the district level in India, pinpointing specific clusters where anaemia among men was particularly concentrated. Moreover, we delved into the factors associated with anaemia at the district level. Utilizing data from the National Family Health Survey-5, our research unearthed crucial findings that warrant immediate policy attention.

Our research reveals that close to 30% of men in rural India suffer from anaemia in 2019–2021. We observed significant geographical disparities at both the state and district levels and identified several hotspots and cold spots of men’s anaemia in the rural areas of the country. Recognizing the significance of this spatial analysis, hotspots become focal points for strategic resource allocation and targeted policies to combat anaemia effectively in regions with considerably high prevalence. Meanwhile, cold spots offer valuable insights as potential models for best practices and interventions, showcasing areas where anaemia rates are notably lower and effective strategies may already be in place. Of particular concern are the four prominent hotspots situated in eastern and central Indian districts. The one spanning across the states of West Bengal, Bihar, Jharkhand, and Odisha is the largest among all. The other hotspots include the Dandakaranya hotspot (southern Chhattisgarh and eastern Odisha) and the one in western Madhya Pradesh bordering the districts of Gujarat and Maharashtra. The analysis further suggests that high anaemia prevalence in these hotspots coincides with higher proportion of low education and high proportion of tribal proportion. These hotspots, characterized by the convergence of high anaemia prevalence with factors such as lower educational attainment and a significant tribal demographic, present a complex challenge. Addressing anaemia in these hotspots necessitates a multifaceted approach with targeted interventions that are specifically tailored to the unique socio-economic and cultural contexts of these areas.

The spatial regression analysis revealed that a number of factors including men’s age, level of education, social group, religion, wealth status, BMI, consumption of alcohol and smoking were found to be significantly associated with anaemia prevalence among rural men at the district level. It was found that prevalence of anaemia increases with the age of men. This could be attributed to the factors that older men are more prone to diseases such as cardiovascular diseases, chronic kidney disease and other non-communicable diseases. These diseases (now significantly prevalent in rural India) aggravate the risk of developing anaemia [32]. Besides, older men in India with diabetes mellitus are associated with higher risks of anaemia [33]. Furthermore, aged men with dementia, disabilities, and emotional problems may struggle to eat, which raises their chance of acquiring anaemia [32, 34]. Previous studies have established the link between age of men and increased risk of anaemia [35]. The map of spatial autocorrelation between percentage of older men and anaemia prevalence reveals that some districts in West Bengals, Assam and some dispersed pockets of central India are evidence of age functioning as significant predictor of anaemia. However, southern Indian districts show high-low cluster (high percentage of aged men and low anaemia) meaning thereby other factors negatively associated with anaemia are more prevalent in those districts.

Our analysis also revealed a statistically significant and positive association between the percentage of men with no education and anaemia in rural India, which is an intriguing discovery in rural context. It was, however, established in previous research although in different context [36]. It is to be noted that men with no or little education have limited access to the necessary resources such as balanced diet resulting into inadequate intake of nutrition. Moreover, it can be challenging for the underprivileged men to have the access to proper healthcare [37, 38]. Compared to their less educated counterparts, individuals with greater educational achievement tend to be healthier and live longer. The gaps are significant and growing and contribute to higher anaemia prevalence in men with no education. The key to eliminating health disparities and enhancing population health is comprehending the conditions in which this link emerges and operates [39–41]. Our results suggest that in rural India, education is a significant predictor of anaemia, and bivariate LISA map clearly demonstrated that a lack of education is an obstruction to anaemia reduction in eastern India. In a similar fashion, the spatial arrangement and clustering of anaemia and lack of schooling among men also revealed that districts in South Indian states, Mizoram, Nagaland, and Uttarakhand, where the percentage of men with no schooling is low, also have a lower prevalence of anaemia.

Our findings suggest that greater percentage of Muslim men population in the districts of West Bengal, Assam and Jammu and Kashmir was associated with high prevalence of anaemia. However, this pattern is not consistent, as several districts in Jharkhand, Odisha, Chhattisgarh, and northern Maharashtra have a higher anaemia prevalence despite a lower percentage of Muslim men. This finding, nonetheless, makes a case for further investigation into the prevalence of anaemia in the Muslim population.

Although none of the spatial regression models, OLS and SLM identified BMI as significant predictor of anaemia prevalence among men, the bivariate LISA map tells a different story. The prevalence of anaemia and the percentage of underweight men were significantly and positively correlated in many districts of West Bengal, Bihar, Jharkhand, and the western portion of Madhya Pradesh. Existing literature suggests that nutritional status has a direct bearing on anaemia [42] and it is strongly associated with being underweight [25, 43]. Malnutrition may not be directly related to anaemia. However, studies found that there is a possibility of underweight individuals having a weakened immune system, which can lead to a variety of health issues such as parasitic infections or chronic inflammation. These factors are in charge of lowering the level of haemoglobin in the blood of underweight individuals [44]. Policies aiming to reduce the burden of anaemia among Indian males through nutritional interventions may find help from our regional-spatial model. It must be, however, highlighted here that several districts in Jammu and Kashmir have a high prevalence of anaemia in men despite a low percentage of underweight men, Therefore, the association between BMI and anaemia must be taken with caution where some other factors (like quality of food and nutrition) that affect anaemia may be at play.

High prevalence of anaemia among men was associated with higher proportion of men belonging to the poorest wealth quintiles in the districts of West Bengal, Jharkhand, Odisha, southern Chhattisgarh, Assam, and western pocket of Madhya Pradesh. Poverty is a significant determinant to poor health and a barrier to receiving necessary medical care. Financial constraints prevent the marginalised from obtaining the necessities for optimal health, such as enough quantity of high-quality food and care [45, 46]. Our findings in relation to anaemia reflect the same association. However, this relationship may have links with other dimensions of poverty, such as a lack of awareness about the best ways to achieve and promote health [47].

The Government of India has put into effect numerous programs and policies aimed at reducing the prevalence of anaemia in the country. Nevertheless, the majority of these initiatives have primarily focused on Women of Reproductive Age (WRA) and children. For example, the National Nutritional Anaemia Prophylaxis Programme (NNAPP) has been distributing free iron and folic acid supplements to children and pregnant women through primary healthcare facilities since its establishment in 1970 [48]. The ‘12-by-12 initiative which was launched in 2007 was focused on young children [10]. The revised NNAPP and the Weekly Iron and Folic Acid Supplementation (WIFS) programme focused on adolescent boys and girls [4, 10]. In 2018, a comprehensive new initiative called ‘*Anaemia Mukt Bharat*’ was introduced in India. This initiative employs multiple strategies to combat anaemia, including iron and folic acid supplementation, deworming, food fortification in schools, and malaria screening. It targets both male and female adolescents aged 10 to 19, as well as adults aged 20 to 49 [4, 10].

It is evident that, until recently, there has been a conspicuous lack of government programs or initiatives explicitly focused on alleviating anaemia among men, regardless of whether they reside in rural or urban areas. Men, especially those in productive age groups, when affected by this condition, may not fully realize their potential, and may experience reduced productivity as a result. This situation could potentially place additional strain on the healthcare system due to the illnesses associated with anaemia. In response, the Indian government must adopt a multifaceted approach to address the high prevalence and spatial disparities in anaemia among men. An initial step towards finding solutions is to implement regular screening of men’s haemoglobin levels to assess the situation. Furthermore, it is essential to thoroughly examine and monitor regional variations in dietary intake and its quality, and take targeted interventions at the micro level. This will help mitigate the spatial disparities in anaemia, particularly among vulnerable rural male populations, as identified in this study.

### Limitations of the study

The current study has a few limitations that should be acknowledged. Firstly, the underlying causes of anaemia were not available within the NFHS dataset, hence we could not include them in this study. Secondly, haemoglobin concentration in NFHS-5 was assessed using the battery-operated portable HemoCue Hb 201 + analyser. However, it is crucial to acknowledge that concerns have been raised by researchers regarding its measurement accuracy. Several studies have indicated that the use of this device might overestimate the prevalence of anaemia, and the results obtained may diverge from those derived through laboratory-based testing methods [26, 49]. Thirdly, it should be noted that in our study, we were unable to assess the relationship between iron intake and anaemia among men due to the unavailability of relevant data. Further research in this area is recommended to explore this association and gain a deeper understanding of its implications. Finally it is crucial to acknowledge that while the data used in this study was orginally collected for generating state-level estimates, we have repurposed it to gain insights into the district-level spatial distribution of anemia. It is important to exercise caution when utilizing these district-level estimates as they may be less precise at times.

## Conclusion

In a country like India where there have been extensive discussions and policy focus on utilising available demographic dividend, it is surprising that research on anaemia among men has received scant attention. This oversight is particularly concerning given the potential implications for the health, well-being, and productivity of the male population. Leveraging mapping and spatial autocorrelation techniques, our research pinpoints high-priority areas that require targeted policymaker attention. Our study revealed a distinct regional divide, with higher anaemia prevalence in the east and lower prevalence in the south among men. We have identified a number of hotspots and cold spots of anaemia prevalence and found several factors such as age, education, religious affiliation, wealth status, underweight status, and regular alcohol consumption associated with anaemia among men in rural India at the district-level. Our findings underscore the importance of formulating policies that recognize the district-level spatial variations in anaemia prevalence and its underlying determinants. Notably, the enduring impact of education and poverty on men’s haemoglobin levels calls for affirmative actions at the district-level, particularly among marginalized populations. Tracking men’s haemoglobin levels should be a routine part of health monitoring and policy formulation. Ultimately, addressing anaemia among men should be considered a pressing public health issue, with interventions designed to account for the social determinants of anaemia among this demographic.

## Data Availability

The data used in this study can be obtained for free by submitting an online request to the Demographic and Health Surveys (DHS) repository https://dhsprogram.com/data/available-datasets.cfm. The base layer for the maps was downloaded as a free GIS file from https://spatialdata.dhsprogram.com/boundaries/#view=table&countryId=IA for national and sub-national boundaries).
